# An Intronic microRNA Links Rb/E2F and EGFR Signaling

**DOI:** 10.1371/journal.pgen.1004493

**Published:** 2014-07-24

**Authors:** Mary Truscott, Abul B. M. M. K. Islam, James Lightfoot, Núria López-Bigas, Maxim V. Frolov

**Affiliations:** 1 Department of Biochemistry and Molecular Genetics, University of Illinois at Chicago, Chicago, Illinois, United States of America; 2 Department of Genetic Engineering & Biotechnology, University of Dhaka, Dhaka, Bangladesh; 3 Department of Experimental and Health Sciences, Barcelona Biomedical Research Park, Universitat Pompeu Fabra (UPF), Barcelona, Spain; 4 Catalan Institution for Research and Advanced Studies (ICREA), Barcelona, Spain; Geisel School of Medicine at Dartmouth, United States of America

## Abstract

The importance of microRNAs in the regulation of various aspects of biology and disease is well recognized. However, what remains largely unappreciated is that a significant number of miRNAs are embedded within and are often co-expressed with protein-coding host genes. Such a configuration raises the possibility of a functional interaction between a miRNA and the gene it resides in. This is exemplified by the *Drosophila melanogaster dE2f1* gene that harbors two miRNAs, *mir-11* and *mir-998*, within its last intron. miR-11 was demonstrated to limit the proapoptotic function of dE2F1 by repressing cell death genes that are directly regulated by dE2F1, however the biological role of miR-998 was unknown. Here we show that one of the functions of miR-998 is to suppress dE2F1-dependent cell death specifically in *rbf* mutants by elevating EGFR signaling. Mechanistically, miR-998 operates by repressing dCbl, a negative regulator of EGFR signaling. Significantly, dCbl is a critical target of miR-998 since dCbl phenocopies the effects of miR-998 on dE2f1-dependent apoptosis in *rbf* mutants. Importantly, this regulation is conserved, as the miR-998 seed family member miR-29 repressed c-Cbl, and enhanced MAPK activity and wound healing in mammalian cells. Therefore, the two intronic miRNAs embedded in the *dE2f1* gene limit the apoptotic function of dE2f1, but operate in different contexts and act through distinct mechanisms. These results also illustrate that examining an intronic miRNA in the context of its host's function can be valuable in elucidating the biological function of the miRNA, and provide new information about the regulation of the host gene itself.

## Author Summary

Animal genomes encode hundreds of microRNA genes that impact all areas of biology by limiting the expression of their targets. What remains largely unappreciated is that a significant proportion of microRNA genes are embedded within protein-coding genes, and are often co-expressed with their hosts, which raises the possibility of a functional interaction between them. The *mir-998* gene is located within an intron of the gene encoding *Drosophila* E2F1 transcription factor. E2F1 can induce the expression of cell death genes, and its activity is negatively regulated by the pRB tumour suppressor protein. In certain settings, unrestrained E2F1 activity is sufficient to induce cell death in cells lacking functional pRB. Here, we show that miR-998 limits cell death in *Rb*-deficient cells by repressing dCbl, a negative regulator of Epidermal Growth Factor Receptor signaling (EGFR). miR-998 also augments EGFR signaling in differentiating photoreceptor cells. Furthermore, we show that the interaction between miR-998 and Cbl is conserved: in human cells, miR-29, a mir-29/998 seed family member, enhances EGFR signaling by targeting c-Cbl. Therefore, by examining the role of an intronic microRNA in the context of its host's function, we identified an important microRNA target and uncovered a biological function of the microRNA.

## Introduction

MicroRNAs (miRNAs) are short non-coding RNAs that regulate the expression of mRNA targets, thereby modulating biological processes including development, proliferation, metabolism, homeostasis and tumorigenesis. While some miRNAs elicit strong effects, many miRNAs operate more subtly to buffer a system or response to a signal. There is significant redundancy among miRNAs of the same family in regulating their target genes, making it difficult to identify the physiological role of an individual miRNA. The absence of strong loss-of-function phenotypes of a significant proportion of miRNAs has significantly hampered the characterization of their functions *in vivo* [1]. A number of approaches have been used to reveal miRNA functions, including combining mutations to generate synthetic phenotypes [2]–[5]. What remains largely unappreciated is that approximately 40% of miRNA genes are embedded within, and frequently co-expressed with protein-coding genes [6], [7]. There is a growing number of examples of intronic miRNAs directly impacting the function of the genes in which they reside [8]–[12]. Therefore, investigating a miRNA in the context of its host gene function could potentially provide insight into the biological roles of a large number of miRNAs. The value of such an approach is illustrated by recent studies of the *Drosophila melanogaster dE2f1* gene.

**Figure 1:**
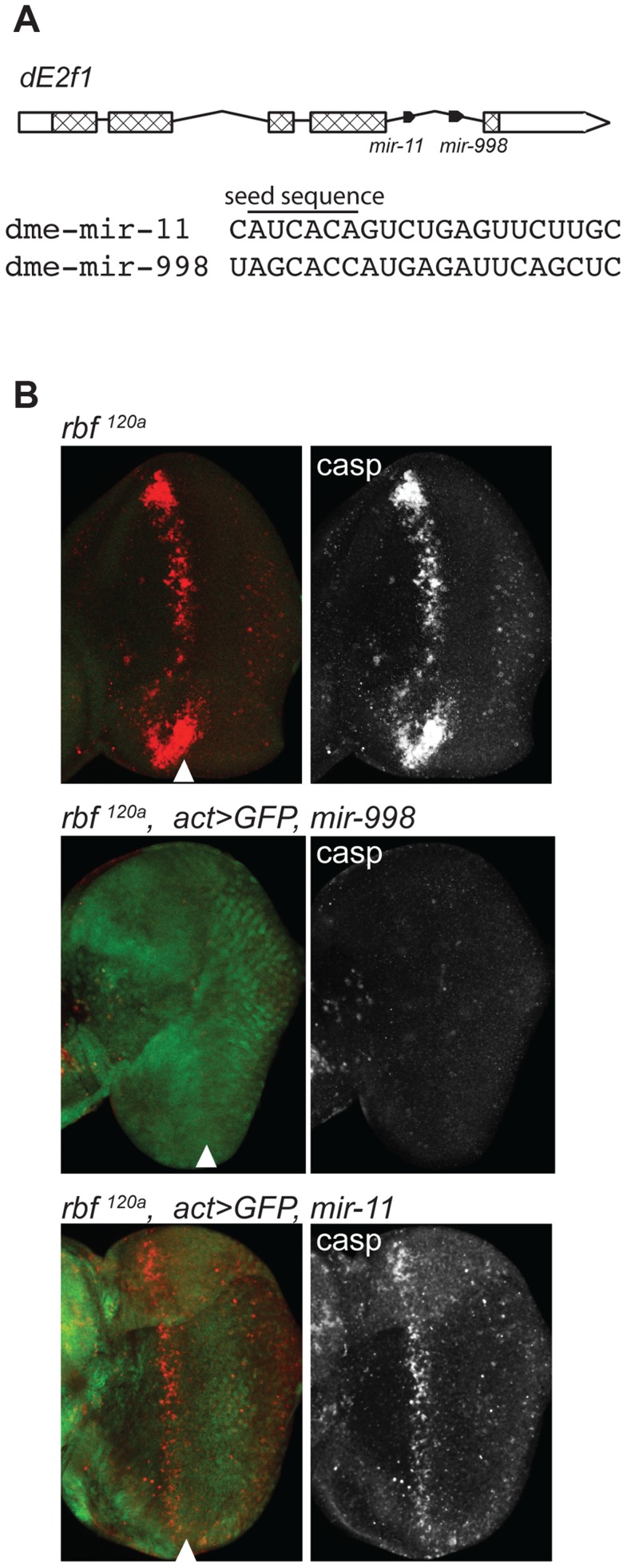
miR-998 limits dE2F1-dependent cell death in *rbf1^120a^* eye discs. (A) Diagram of the *dE2f1* transcript. Two miRNAs are in the last intron: *mir-11* and *mir-998*, and are co-transcribed with *dE2f1*. See also Figure S1. The aligned sequences of mature miR-11 and miR-998 are shown, with the seed sequence highlighted. (B) Third instar larval eye discs were immunostained with an antibody that recognizes active caspases in dying cells (C3 antibody). GFP and miR-998 or miR-11 were expressed in the entire eye disc of *rbf1^120a^* hemizygous males using a flip-out technique induced by ey-FLP. Full genotypes are: *rbf1^120a^*, *ey-FLP*; +/+ (top), *rbf1^120a^*, *ey-FLP; act5c>CD2>GAL4, UAS-GFP/UAS-mir-998* (middle), and *rbf1^120a^*, *ey-FLP; act5c>CD2>GAL4, UAS-GFP/UAS-mir-11* (bottom).

The dE2f1 transcription factor, and its mammalian homologs coordinate the expression of genes involved in cell proliferation and cell death. In a variety of systems, E2F is rate-limiting for S phase entry while it triggers apoptosis in specific contexts. The last intron of the *Drosophila* E2F gene *dE2f1* harbors a miRNA, *mir-11*, which is co-expressed with *dE2f1* (Figure 1A and Figure S1). The loss of *mir-11* was shown to strongly enhance dE2F1-dependent DNA damage-induced apoptosis even though it was insufficient to cause cell death in unprovoked settings. Therefore, the physiological role of *mir-11* was revealed only when examined in the sensitized background of its host gene. This function of miR-11 is explained by its ability to directly regulate the expression of dE2F1-regulated cell death genes, thus highlighting a complex interaction between an intronic miRNA and its host gene [1], [8]. In addition to *mir-11*, the last intron of the *dE2f1* gene contains another miRNA, *mir-998*. The sequence of mature miR-998 is different than that of miR-11, particularly at the 5′ end in the seed sequence (Figure 1A), which is the primary determinant of miRNA target selection. Therefore the two miRNAs are likely to regulate distinct sets of genes and, consequently, may have different functions. However since miR-998 was only recently identified, nothing was known about its biological function. Here we show that miR-998 limits dE2F-dependent cell death, but it does so in a different context and by a different mechanism than miR-11. While miR-11 repressed components of the core cell death machinery, including *rpr* and *hid*, miR-998 limited E2F-dependent cell death by elevating prosurvival signaling downstream of the Epidermal Growth Factor Receptor (EGFR) through regulation of *dCbl*, a negative regulator of EGFR. Thus, our data reveal a novel layer of intrinsic regulation at the *dE2f1* genomic locus involving intronic miRNAs.

## Results

### miR-11 and miR-998 have different functions

Rbf is a negative regulator of dE2F1. The loss of *rbf* sensitizes cells to dE2F1-dependent apoptosis. As has been previously shown, there is a high level of apoptosis in a band running along the anterior edge of the morphogenetic furrow of *rbf* mutant eye discs [2]–[5], [13]. This stripe of apoptotic cells can be revealed by staining with the C3 antibody, which specifically recognizes activated caspases (Figure 1B). Importantly, apoptosis is dependent on dE2F1 since it was suppressed in *rbf*, *dE2f1* double mutant animals [6], [7], [13]. To examine the effect of miR-998, we expressed a *UAS-mir-998* transgene in the developing eyes of *rbf* mutant animals using the *ey-FLP*; *Act*≫*Gal4* (Flip-out) system. Remarkably, no apoptotic cells were found in *rbf^120a^*, *act>mir-998* eye discs, indicating that miR-998 strongly suppressed E2F-dependent cell death in this context (Figure 1B). Interestingly, unlike miR-998, miR-11 failed to block apoptosis in *rbf* mutants as the number of C3 positive cells was similar between *rbf^120a^*, *act>mir-11* and *rbf^120a^* eye discs.

Differences in suppression of cell death in *rbf* mutant cells by miR-11 and miR-998 prompted us to investigate the impact of the two miRNAs on dE2F1-dependent apoptosis in other settings. When dE2F1 expression is driven by the *Act88F-Gal4* driver, high levels of apoptosis in the wings of newly eclosed adults give rise to gnarled, blistered wings that have a downward curvature [8]–[12], [14]. This cell death phenotype is strongly rescued by miR-11 (Figure S2A and [8]). However, the wings of *Act88F>dE2f1, mir-998* animals were indistinguishable from the wings of *Act88F>dE2f1* adults, suggesting that expression of miR-998 was insufficient to suppress apoptosis (Figure S2A). Next, we performed genetic interaction tests in the eye imaginal disc. Ectopic expression of *dE2f1* in the posterior compartment of the eye imaginal disc potently induces apoptosis [15]. This apoptosis is strongly suppressed by co-expression of miR-11 (Figure S2B and [8]). In contrast, miR-998 had no effect on E2F1-induced cell death in the posterior compartment, as the level of C3 staining was indistinguishable between *GMR>dE2f1/dDP/mir-998* and *GMR>dE2f1/dDP* eye discs (Figure S2B). In addition to apoptosis, *GMR>dE2f1/dDP* had been shown to induce unscheduled proliferation that can be visualized by BrdU labeling [15]. However, neither miR-11 nor miR-998 modulated dE2F1-induced proliferation, as the level of E2F1-induced ectopic BrdU incorporation was largely unchanged by co-expression of miR-11, as was previously shown [8], or miR-998 (Figure S2B).

Therefore we concluded that overexpression of miR-998 suppressed dE2F1-dependent apoptosis in *rbf* mutants but not when dE2F1 was ectopically expressed in the eye or in the wing. In contrast, miR-11 suppressed dE2F1-induced phenotypes but failed to block apoptosis in *rbf* mutants. Thus, miR-998 and miR-11 both suppressed E2F-dependent cell death, but did so in mutually exclusive contexts.

### Loss of *mir-998* enhances apoptosis in *rbf* mutants

**Figure 2:**
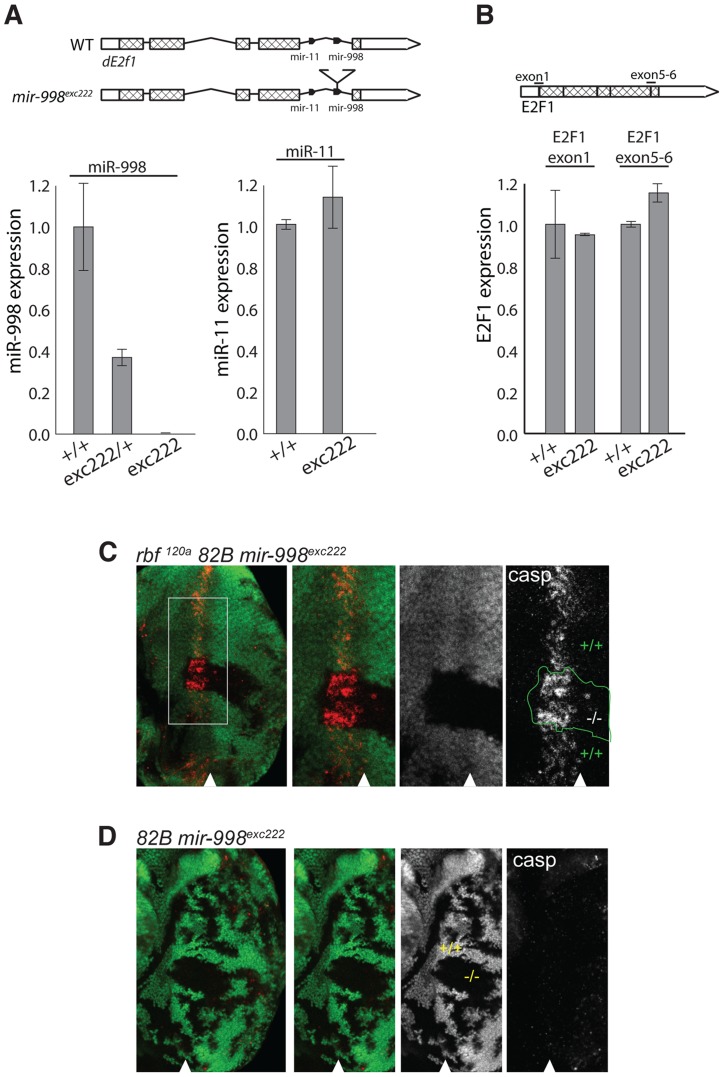
*mir-998* ^exc222^ mutant allele enhances apoptosis in *rbf* mutant eye discs. (A) cDNA was prepared from RNA extracted from +/+ (Canton S, wild-type), *mir-998* ^exc222^/+, or *mir-998* ^exc222^ adult fly heads. The expression of *mir-998* and *mir-11* were measured by Taqman assay, and normalized to *β-tubulin* levels. Expression in +/+ was designated as 1.0 and the expression in *mir-998* ^exc222^was compared. (B) cDNA was prepared from RNA extracted from +/+ (Canton S, wild-type) or *mir-998* ^exc222^ third instar larvae. The expression of *de2f1* mRNA was measured using qPCR, and normalized to *β-tubulin* levels. Expression in +/+ was designated as 1.0, and the expression in *mir-998* ^exc222^was compared. E2F1 exon1 primers span the translation start site and E2F1 5–6 primers span the exon 5-exon 6 junction. (C and D) Clones of *mir-998* mutant tissue were generated using *ey-FLP* in *rbf ^120a^* hemizygous (top), or *rbf* wild-type (bottom) animals. The full genotypes are *rbf1^120a^*, *ey-FLP/Y*; *FRT 82B mir-998^exc222^/82B GFP* (C) and *ey-FLP/+*; *FRT 82B mir-998* ^exc222^/*82B GFP* (D). Third instar larval eye discs were dissected, fixed, and stained with an antibody recognizing active caspase (casp). The full disc is shown in the left panel, while a higher magnification in panels on the right. Mutant clones were outlined and wild-type *mir-998* was indicated by +/+, and −/− for *mir-998^exc222^*. A minimum of 10 larvae were analyzed for each genotype, and representative results are shown.

The results described above using a miR-998 transgene raise the question of whether endogenous miR-998 operates in a similar manner and blocks apoptosis in *rbf* mutants. Therefore we examined the consequence of the loss of *mir-998* in the background of the *rbf^120a^* mutation. Since there were no pre-existing *mir-998* mutants, we generated a *mir-998* null allele (for details see Materials and Methods and Figure S3). It was essential that the *mir-998* loss-of-function allele did not disrupt the expression of *mir-11* or *dE2f1*, such that any observed phenotype could be attributed specifically to the function of miR-998. We used one *piggyBac* element and two *P* elements inserted near the *dE2f1* gene and screened for local transpositions of these transposons into intron 5, which harbors miR-11 and miR-998. Out of 4,254 transposition events a single *P* element insertion into intron 5 was recovered and then used to screen for imprecise excisions that specifically disrupted *mir-998*. Among 400 excision events only two imprecise events were isolated and both of them retained a small piece of the *P*-element. The *mir-998^exc222^* allele contained an 87 bp insertion within the *mir-998* hairpin that is expected to disrupt the correct folding and processing of miR-998. Indeed, no mature miR-998 was detected in whole 3^rd^ instar larvae, larval eye imaginal discs, or adult heads from *mir-998^exc222^* mutant animals (Figure 2A). Importantly, the expression of *dE2f1* and miR-11 were not affected in the *mir-998^exc222^* mutant animals (Figure 2B). We concluded that *mir-998^exc222^* is a null allele of *mir-998*.

Clones of *mir-998^exc222^* homozygous mutant cells in *rbf* mutant eye discs were generated using the *ey*-Flp/FRT system and apoptotic cells were visualized with the C3 antibody. The *mir-998* mutant tissue was marked by the absence of GFP, while tissue that contained a wild-type *mir-998* allele expressed GFP. Since the entire disc was mutant for *rbf*, cell death occurred in the distinctive pattern in the morphogenetic furrow in both *mir-998* mutant and *mir-998* wild type tissue. However, significantly elevated C3 staining was consistently observed in clones of *mir-998^exc222^* mutant cells compared to adjacent *mir-998* wild-type tissue (Figure 2C). In contrast, no apoptosis was detected in the furrow when clones of *mir-998* mutant cells were induced in wild type eye discs (Figure 2D). Thus, the loss of *mir-998* specifically sensitized *rbf* mutant cells to apoptosis, while overexpression of miR-998 blocked cell death in *rbf* mutants.

### miR-998 modulates EGFR signaling

How does miR-998 suppress apoptosis of *rbf* mutant cells? The specific pattern of apoptosis in *rbf* mutant eye discs is due to the coincident transient reduction of EGFR signaling in the morphogenetic furrow that, in turn, lowers prosurvival cues. As a result, the level of unrestrained dE2F1 becomes sufficient to trigger cell death in this region of *rbf* mutant but not in wild type eye discs [13]. In addition, the cell death in *rbf* mutants was shown to be dependent on the expression of the pro-apoptotic genes *reaper* (*rpr*) and *hid*. Therefore we examined impact of miR-998 on EGFR signaling and on *hid* and *rpr*.

**Figure 3:**
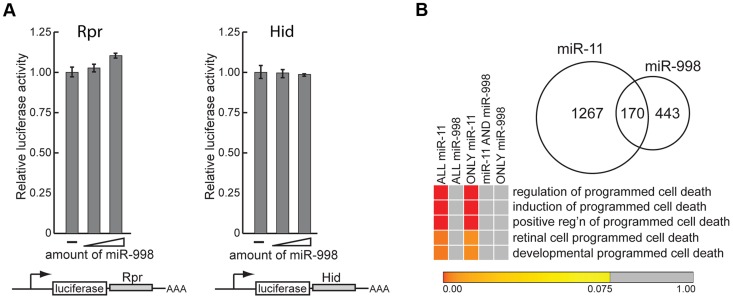
miR-11 and miR-998 limit dE2F-dependent cell death through different targets. (A) 3′ UTR sensor assays were performed in HeLa cells using *rpr* and *hid* 3′ UTR sensors. The indicated 3′ UTR sensor plasmid was transfected with increasing amounts of pcDNA3/*mir-998* plasmids. Cells were harvested 40–48 h post-transfection, and *Renilla* and *Firefly* luciferase activities were measured. (B) Comparison of predicted miR-11 and miR-998 targets (see Table S1 for lists of predicted miR-11 and miR-998 targets). Heat map of GOBP enrichment analysis of predicted miR-11 and miR-998 targets (FDR <or = 0.075).

We began by determining whether miR-998 regulates expression of *rpr* and *hid* in luciferase sensor assays. The 3′UTRs of *rpr* and *hid* were cloned downstream of the constitutively expressed luciferase gene. Increasing amounts of miR-998 were co-transfected with *rpr* or *hid* 3′UTR sensors, and luciferase activity was measured. As shown in Figure 3A, expression of miR-998 did not modulate *rpr* or *hid* 3′ UTR reporters. To corroborate this result we compiled a list of predicted miR-998 targets and performed Gene Ontology of Biological Processes (GOBP) enrichment analysis. Interestingly, none of the GOBP terms associated with apoptosis were statistically enriched among miR-998 targets (Figure 3B, Table S2). In contrast, as it has been shown previously [8], GOBP terms that relate to the induction and positive regulation of cell death were significantly enriched among miR-11 targets (Figure 3B, Table S2). Furthermore, although miR-11 and miR-998 share 170 common targets, cell death GOBP terms were not enriched among them but were overrepresented among genes that are exclusively miR-11 targets. Thus, the sensor assays and bioinformatics analyses do not support the explanation that suppression of apoptosis in *rbf* mutants by miR-998 occurs through the direct regulation of cell death genes.

**Figure 4:**
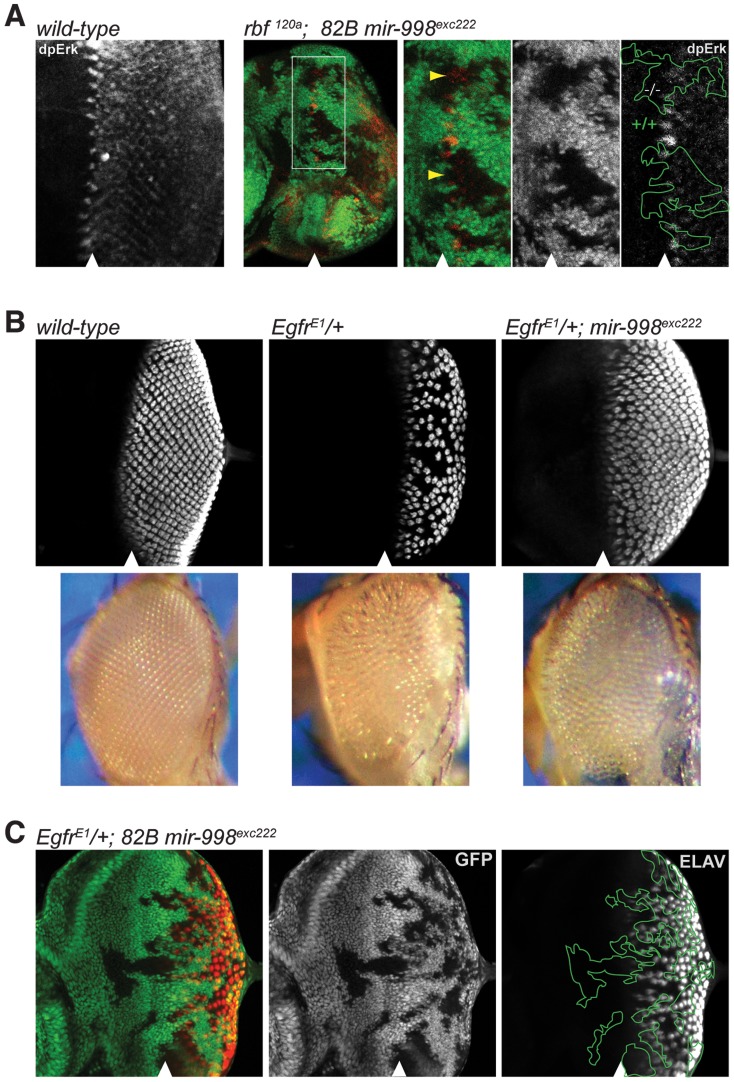
*mir-998^exc222^* suppresses EGFR signaling. (A) Third instar larval eye discs were dissected, fixed, and stained with an antibody recognizing active Erk (dpErk). A wild-type larval eye disc is shown in the left-most panel. To the right: clones of *mir-998^exc222^* mutant tissue were generated using *ey-FLP* in *rbf ^120a^* hemizygous animals as in Fig. 2C. The full disc as well as a higher magnification are shown. Mutant clones were outlined and wild-type *mir-998* was indicated by +/+, and −/− for *mir-998^exc222^*. Yellow arrowheads show regions in *mir-998* mutant tissue with significantly less dpERK staining than expected. (B) Third instar larval eye discs were harvested from wild-type control, *Egfr^Elp^*/+, and *Egfr^Elp^*/+; *82B mir-998^exc222^* animals. Dissected tissue was fixed and stained with an antibody recognizing ELAV (red), which is expressed in photoreceptor clusters. Below are images of adult eyes from wild-type control, *Egfr^Elp^*/*+*, and *Egfr^Elp^*/+; *82B mir-998^exc222^* animals. Images were taken at the same magnification. (C) Third instar larval eye discs were harvested from *ey-FLP*; *Egfr^Elp^*/+; *82B mir-998^exc222^*/*82B GFP* animals. Tissue that is wild-type for *mir-998* is marked by GFP (green), while clones of *mir-998* mutant tissue are marked by the absence of GFP. Mutant clones were outlined and wild-type *mir-998* was indicated by +/+, and −/− for *mir-998^exc222^*. Analysis was performed on a minimum of 10 larvae of each genotype. The position of the morphogenetic furrow is marked with an arrowhead.

Next, we asked whether EGFR activity is altered in *mir-998* mutants. The level of EGFR activity is accurately reflected by di-phosphorylated, activated ERK (dpERK) [16]. During eye development, EGFR signaling is transiently reduced within the morphogenetic furrow, while EGFR activity is high in groups of cells that form the ommatidial preclusters in a column immediately posterior to the morphogenetic furrow, which is revealed by the dpERK antibody. Within a column, clusters are specified sequentially in short intervals beginning at the midline. This gives rise to a gradual rise and fall of dpERK staining within a column (Figure 4A, left panel). To examine the level of EGFR signaling in *mir-998* mutants, clones of *mir-998^exc222^* mutant cells were generated in *rbf^120a^* mutant eye discs and stained with the dpERK antibody. While the pattern of natural variation of dpERK staining was not altered in *mir-998* mutant tissue, the intensity of dpERK staining was reduced within clones of mir-998 mutant cells, as well as in wild type tissue immediately adjacent to the clonal boundary (Figure 4A, see yellow arrowheads). Since the level of dpERK expression reflects the level of EGFR signaling, this indicated that the loss of *mir-998* reduces EGFR activity and this may explain the enhancement of apoptosis in *rbf* mutants.

EGFR signaling is used reiteratively throughout development, including in the recruitment of photoreceptor cells into the ommatidial clusters of the developing larval eye [17]–[19]. To confirm that EGFR signaling is reduced in *mir-998* mutants, we performed genetic interaction tests between the *mir-998* mutant allele and the dominant gain-of-function *Ellipse* (*Elp*) allele of the *Egfr* gene. The number of ommatidial clusters is significantly reduced in *Egfr^Elp^*/+ larval eye discs as revealed by staining with an ELAV antibody that marks differentiated photoreceptors (Figure 4B and [18], [20]). Strikingly, the *mir-998^exc222^* mutation strongly suppressed this phenotype: there was a dramatic increase in the number of ommatidial clusters in *Egfr^Elp^*/+, *mir-998^exc222^* double mutant eye discs compared to *Egfr^Elp^*/+ single mutant eye discs (Figure 4B). Consistently, the small, rough eye phenotype of *Egfr^Elp^*/+ adult flies was suppressed by the loss of *mir-998* (Figure 4B). To determine whether the effect of *mir-998* is cell autonomous, we generated clones of *mir-998^exc222^* mutant cells in heterozygous *Egfr^Elp^*/+ eye discs. As shown in Figure 4C, the *Egfr^Elp^* mutant phenotype was partially suppressed in cells that were immediately adjacent to the *mir-998^exc222^* mutant tissue suggesting some non-cell autonomous effects. Therefore, the loss of *mir-998* suppresses the EGFR gain-of-function phenotype, which is consistent with the reduction of dpERK activity (Figure 4A) and therefore EGFR signaling in *mir-998* mutant tissue.

### *dCbl* is downregulated in *mir-998* mutant eye discs, phenocopies the response of the *mir-998* mutant to E2F-dependent apoptosis, and can be directly repressed by miR-998 *in vitro*

To gain insight into the molecular basis of the genetic interaction between miR-998 and EGFR signaling, we asked whether the loss of *mir-998* results in misexpression of gene(s) that are known to be connected to the EGFR signaling pathway. To identify such genes in an unbiased manner we performed gene expression microarrays using RNA isolated from *mir-998^exc222^* homozygous mutant and wild type eye discs. This analysis led to identification of a set of 382 genes that were differentially expressed (DE) in the *mir-998* mutant. The list of DE genes was then mined for genes with terms related to the EGFR signaling pathway by referring to AmiGO and KEGG pathway databases, as well as the literature [21]–[23] (Table S3).

**Figure 5:**
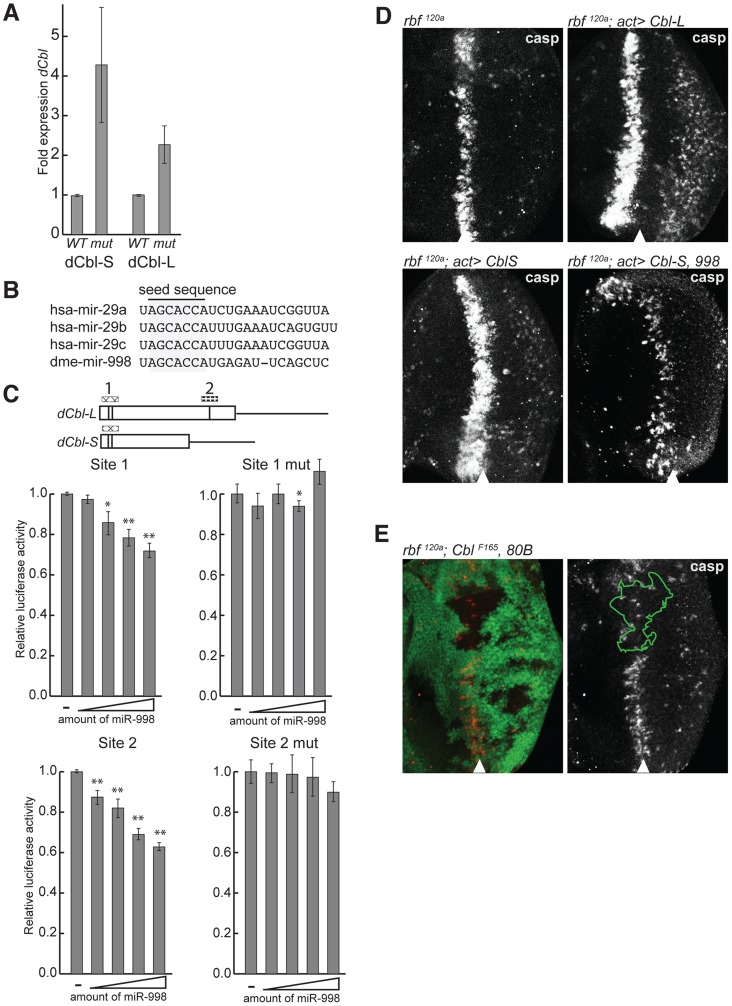
miR-998 enhances EGFR signaling by repressing *dCbl*. (A) cDNA was prepared from RNA extracted from +/+ (Canton S, wild-type) or *mir-998* ^exc222^ third instar larval eye discs. The expression of *dCbl-S*, and *dCbl-L* were measured using qPCR, and normalized to *rp49* or *β-tubulin* levels. Expression in +/+ was designated as 1.0 and the expression in *mir-998* ^exc222^ was compared. (B) Alignment of mature miRNA sequences of mir-29 seed family homologs. Seed sequences are highlighted. (C) 3′ UTR sensor assays were performed in HeLa cells using sequences predicted to be regulated by miR-29 or miR-998, or sequences carrying mutations in miR-998 binding sites. The indicated 3′ UTR sensor plasmid was transfected with pcDNA3/empty or increasing amounts of pcDNA3/*mir-998* plasmid. Cells were harvested 24–48 h post-transfection, and luciferase activities were measured. A minimum of three independent transfections was performed for each sensor construct. Error bars represent standard error. Asterisks are shown for differences between vector and miR-998 that are statistically significant by t-test (* is p<0.05, ** is p<0.01). (D) Third instar larval eye discs were immunostained with an antibody that recognizes active caspases in dying cells. GFP and *dCblS*, *dCblL*, or *miR-998* were expressed in the entire eye disc of of males hemizygous for *rbf1^120a^* using a flip-out technique induced by ey-FLP. Full genotypes are: *rbf1^120a^*, ey-FLP; +/+ (top left), *rbf1^120a^*, *ey-FLP; act5c>CD2>GAL4, UAS-GFP/UAS-dCblL* (top right), and *rbf1^120a^*, *ey-FLP; act5c>CD2>GAL4, UAS-GFP; UAS-dCblS* (bottom left), and *rbf1^120a^*, *ey-FLP; act5c>CD2>GAL4, UAS-GFP/UAS-miR-998; UAS-dCblS* (bottom right). (E) Third instar larval eye discs from *rbf1^120a^*, *ey-FLP/Y*; *dCbl^F165^*, FRT 80B/GFP, FRT 80B animals were dissected, fixed, and stained with an antibody recognizing active caspase (casp).

Of 80 genes associated with the EGFR pathway, only one was significantly differentially expressed in the *mir-998^exc222^* microarray: *dCbl-S*. dCbl negatively regulates signaling from EGFR by binding to the activated, phosphorylated receptor, and inducing its ubiquitination and endocytosis [24]–[27]. According to the microarray data, the expression of *dCbl-S* was increased 3.6-fold in the absence of *mir-998*, which made it a good candidate as a target of repression by miR-998, the prevailing mechanism of miRNA function. To confirm the results of the gene expression microarray, *dCbl* expression was measured in *mir-998* homozygous mutant eye discs by quantitative RT-PCR (qRT-PCR). The *dCbl* gene encodes two dCbl proteins generated from alternatively spliced transcripts which, like their mammalian Cbl homologs, both negatively regulate EGFR signaling in *Drosophila* [28], [29]. As shown in Figure 5A, both *dCbl* transcripts were significantly upregulated in *mir-998^exc222^* eye discs (*dCbl-S*: 4.4-fold, *dCbl-L*: 2.3-fold). Together with the results of genetic interaction tests described above, this suggested a model where miR-998 represses dCbl, a negative regulator of EGFR signaling, to enhance signaling downstream of the EGF receptor.

This model was tested in three sets of experiments. First, we asked whether *dCbl* is a miR-998 target. Since *dCbl* was not identified as a target of miR-998 by bioinformatic prediction, we asked whether the mammalian ortholog, c-Cbl, was a predicted target of the mammalian miRNA members of the mir-29/998 miRNA seed family: mir-29a-c [30] (Figure 5B and Table S1). Analysis revealed that c-Cbl contains five predicted miR-29 target sites including two sites in tandem within a highly conserved region that encodes the tyrosine kinase-binding (TKB) domain. Further analysis of *dCbl* using the RNA22 prediction algorithm with low stringency parameters [31] revealed a putative target site for miR-998 in *dCbl-L* (Figure 5C). The functionality of these sites was then tested in luciferase sensor assays. The identified predicted target sites for miR-998 in *dCbl* were cloned downstream of the *Renilla* luciferase gene, and were transfected with increasing amounts of a miR-998 expression plasmid. Significantly, miR-998 exhibited dose-dependent repression of luciferase 3′UTR sensor constructs carrying either the double miR-29 target site within the highly conserved TKB domain (site 1), or the miR-998 site near the 3′ end of the dCbl-L coding sequence (site 2) (Figure 5C). Moreover, the repression of site 1 and site 2 was completely blocked when the target sequences were mutated. Thus, miR-998 directly repressed luciferase sensor constructs carrying dCbl target sequences. While our sensor assay results are consistent with the notion that miR-998 directly represses dCbl in eye discs, we acknowledge the possibility that miR-998 represses dCbl indirectly through a different mechanism *in vivo*.

Since *dCbl* is a negative regulator of EGFR signaling, its elevated expression in *mir-998* mutants may explain the increased apoptosis in the morphogenetic furrow of *rbf*, *mir-998* double mutants. To test this idea, we examined the effect of *dCbl* overexpression on cell death in *rbf* mutant eye discs. When dCbl-S or dCbl-L were expressed in *rbf^120a^* mutant eye discs using the Flip-out system, a wider band of C3 antibody staining was detected than in the control *rbf^120a^* mutant eye disc. Importantly, co-expression of miR-998 blocked the increase in cell death induced by dCbl, which is consistent with the notion that miR-998 can repress dCbl expression *in vivo* (Figure 5D). In the converse experiment, clones of *dCbl* mutant cells were generated in *rbf^120a^* mutant background and mosaic eye discs were stained with the C3 antibody. Consistent with results of dCbl overexpression described above, the loss of *dCbl* strongly suppressed apoptosis in *rbf* mutants as the number of C3 positive cells was dramatically reduced in the *dCbl* mutant tissue (Figure 5E). Therefore miR-998 represses dCbl, a negative regulator of EGFR signaling that is functionally important for triggering cell death, in the morphogenetic furrow of *rbf* mutant eye discs. Furthermore, dCbl is a critical target of miR-998 in modulating apoptosis in *rbf*-deficient cells since overexpression of dCbl in eye discs mimics the *mir-998* mutant phenotype, while the *dCbl* mutant mimics the miR-998 overexpression phenotype.

### miRNA-dependent repression of dCbl is conserved in mammalian cells

**Figure 6:**
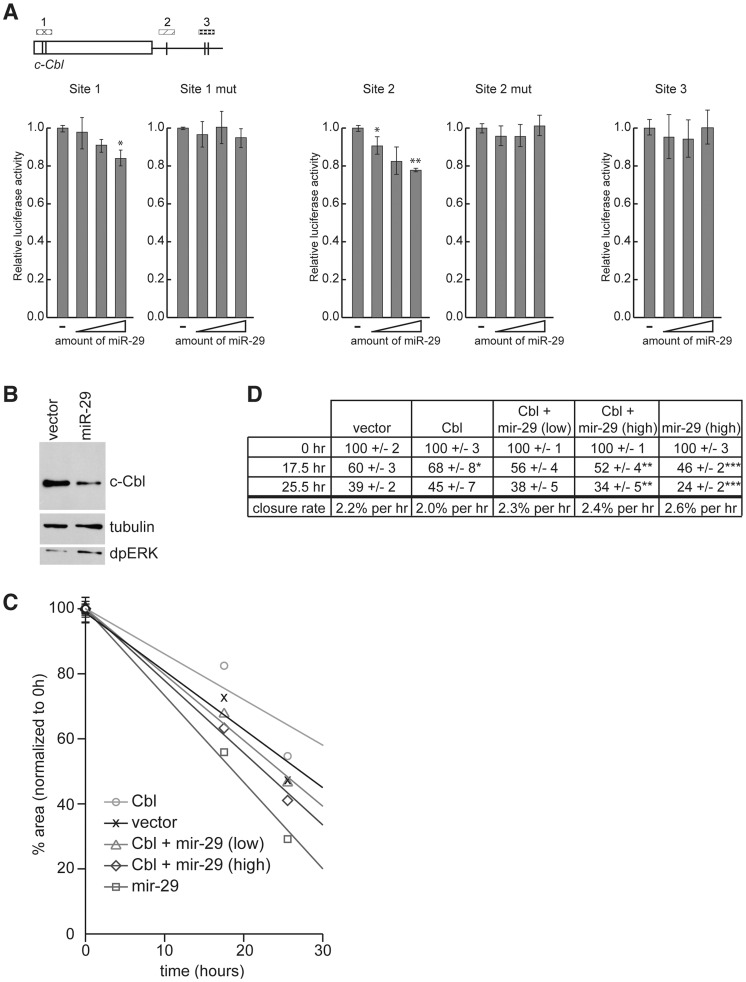
c-Cbl is a target of hsa-miR-29 in mammalian cells. (A) 3′ UTR sensor assays were performed in HeLa cells using sequences predicted to be regulated by miR-29. The indicated wild-type or mutant 3′ UTR sensor plasmids were transfected with pcDNA3/empty alone, or increasing amounts of pcDNA3/*mir-29* plasmids. Cells were harvested 24–48 h post-transfection, and luciferase activities were measured. A minimum of three independent transfections was performed for each sensor construct. Error bars represent standard error. Asterisks are shown for differences between vector and miR-29 that are statistically significant by t-test (* is p<0.05, ** is p<0.01). (B) Cell lysates were prepared from HeLa cells transiently transfected with pcDNA3/empty or pcDNA3/*mir-29* plasmids. The expression of c-Cbl, dpERK, and tubulin were detected by Western blot. (C and D) Wound healing assays were performed on HeLa cells transfected with c-Cbl, miR-29a, or an empty vector control. The area of the wound was measured at each time point and normalized to 0 h. Eight wound areas were measured for each sample at each time point. (C) Average wound areas relative to the initial scratch were plotted for each transfection condition. (D) Summary of scratch assay data. Shown are average wound area and standard error for each transfection condition, and the rate of wound closure (change in % scratch area/time) represented in (C) as trendlines. Asterisks are shown for differences between sample and vector control that are statistically significant by t-test (* is p<0.25, ** is p<0.15, and *** is p<0.005).

miR-998 is part of the mir-29 seed family of miRNAs, which is defined by having identical seed sequences ([30] and Figure 5B). The presence of multiple miR-29 target sites in mammalian c-Cbl raises the question of whether miRNA-dependent regulation of dCbl is conserved in mammals. To address this question we generated and expressed three different luciferase sensors carrying single, or paired predicted miR-29 target sites from the human c-Cbl gene, along with increasing amounts of a miR-29a expression plasmid (Figure 6A). While some miRNAs exert strong repression of their targets, others including miR-29 have been shown to elicit more modest effects in sensor assays and *in vivo* [32]–[36]. Indeed, miR-29a exerted modest but clearly dose-dependent repression of a sensor carrying two sites in tandem in the TKB domain near the 5′ end of the CDS (site 1). miR-29a also repressed a luciferase sensor carrying a predicted miR-29 site in the 3′UTR (site 2), while a sensor carrying tandem sites near the 3′ end of the 3′UTR failed to respond to miR-29 (site 3) (Figure 6A). To address the specificity of repression, we introduced mutations into the mir-29 target sequences in the c-Cbl site 1 and site 2 luciferase sensors and found that miR-29 did not repress these mutant sensors, regardless of the amount of transfected miR-29. Therefore, even though miR-29 modestly repressed sensors containing site 1 and site 2, this repression was specific since the response to miR-29 was dose-dependent, while mutating the sites completely blocked the effect of miR-29. We concluded that miR-29 seed family members can directly target c-Cbl through corresponding paired sites that are present within the highly conserved region in both *Drosophila* and human genes encoding the TKB domain. In addition, *Drosophila* and human miR-29 seed family members can regulate Cbl sensors through distinct target sites that are not conserved between the two species (Figure 5C and Figure 6A).

Having established the functionality of putative miR-29 target sites in the c-Cbl sequence using sensor assays, we asked whether the endogenous c-Cbl could be repressed by miR-29. HeLa cells were transfected with a miR-29 expression plasmid or an empty vector and the level of c-Cbl was analyzed by Western blot analysis 48 hr after transfection. As shown in Figure 6B, the expression of endogenous c-Cbl protein was significantly reduced in cells expressing miR-29 compared to the vector control. Importantly, downregulation of c-Cbl was accompanied by an elevated level of di-phosphorylated ERK (dpERK), which reflects an increase in EGFR/MAPK activity, in response to miR-29a expression.

Previous work showed that in the absence of functional Cbl, the sensitivity of cells to signals from the extracellular milieu is enhanced, and cells exhibit increased growth factor-induced motility in wound-healing scratch assays. The cell migration is mediated in part by ERK-MAPK signaling [37]–[39]. Therefore we tested whether reduction of c-Cbl by miR-29a expression alters the rate of wound-healing in scratch assays. Scratch assays were performed on HeLa cells transfected with constructs expressing miR-29a, c-Cbl, c-Cbl and miR-29a, or an empty vector control. The area of the wound was measured 0, 17.5, and 25.5 hours after the scratch was introduced (Figure 6C and 6D). As expected, cells expressing c-Cbl exhibited decreased motility and delayed wound closure compared to the control. In contrast, expression of miR-29a significantly increased the rate of wound healing compared to both cells expressing c-Cbl, and cells transfected with empty vector. miR-29a also increased the rate of wound healing in cells transfected with c-Cbl, which was consistent with the notion that miR-29 directly represses c-Cbl expression and, as a consequence, its function *in vivo*. 25.5 hours after the scratches were introduced, cells expressing miR-29a had closed all but 24% of the original area of the scratch wound, while the scratch wound of controls cells occupied 39% of the original area, and the scratch in cells expressing c-Cbl was 55% of the original area (Figure 6C and 6D). Co-expression of lower and higher amounts of miR-29 with c-Cbl led to scratches that were 38% and 34% the original area after 25.5 hours, suggesting that miR-29 can suppress the function of its target *in vivo*.

From these experiments we concluded that c-Cbl expression is modulated by miR-29a in mammalian cells and that miR-29a represses endogenous c-Cbl expression. Importantly, repression of c-Cbl expression is accompanied by increased ERK-MAPK signaling and an elevated rate of growth factor-regulated cell migration.

## Discussion

The potential for complex interactions between intronic miRNAs and their host is illustrated by the *Drosophila dE2f1* gene, and the two miRNAs embedded in its last intron: *mir-11* and *mir-998*. We previously showed that *mir-11* directly represses a subset of apoptotic genes that are transcriptional targets of the host gene *dE2f1*, and in doing so, miR-11 limits E2F-dependent cell death induced by DNA damage. Here, we identified a novel layer of regulation in the *dE2f1* locus as miR-998 enhances EGFR cell survival signaling, thereby suppressing E2F-dependent cell death in *rbf* mutant animals. Therefore, the proapoptotic function of dE2F1 is under intrinsic control by two distinct and complementary mechanisms.

Many miRNAs elicit relatively weak effects and therefore their mutant phenotypes are rather subtle. Therefore it is not surprising that both the *mir-11* and *mir-998* mutant alleles were viable, and exhibited no phenotypes on their own. The lack of a mutant phenotype represents a major hurdle in identifying the physiological function of a miRNA [2], [40]. A number of approaches have been taken to reveal miRNA functions such as generating compound mutants, and analyzing mutant phenotypes in the context of disruptions to core regulatory pathways [3]. In our work we have used a different strategy and investigated miRNAs in the context of the function of their host gene. This novel approach turned out to be highly informative and allowed us to identify the elusive functions of two intronic miRNAs embedded within the *dE2f1* gene. Notably, both miRNAs exhibited phenotypes only in E2F-sensitized backgrounds but lacked phenotypes on their own. One implication of our work is that the functions of intronic miRNAs can be linked to their host gene, and where known, the host gene function can be exploited to uncover the physiological roles of embedded miRNAs. This idea is particularly relevant given that approximately 40% of all miRNAs are embedded in protein-coding genes and therefore such approach can be applicable beyond the *dE2f1* locus.

In various systems, inactivation of *Rb* provides a cellular context to investigate E2F-dependent apoptosis. Interestingly, animal models revealed that not every *Rb* mutant cell is equally sensitive to apoptosis. In the *Drosophila rbf* mutant eye disc, apoptosis occurs in a highly reproducible pattern that is determined by the level of prosurvival signaling from the EGF receptor [13]. Our data show that the loss of *mir-998* enhanced cell death in *rbf* mutant eye discs but did not alter the overall pattern of apoptosis. Notably, we did not find evidence that miR-998 directly repressed cell death genes. Therefore, miR-998 is unlikely to function by altering the expression of apoptosis genes. Rather, our results support a scenario where miR-998 represses cell death in *rbf* mutants by enhancing pro-survival EGFR signaling. Using genome-wide approaches we identified *dCbl* as a highly upregulated gene in *mir-998* mutant eye discs. Genetic interaction tests demonstrated that *dCbl* phenocopies the effect of *mir-998* on dE2f1-dependent cell death in *rbf* mutants. Therefore *dCbl* behaves as a critical player that mediates the effect of *mir-998* on apoptosis. We acknowledge that our data do not rule out the possibility that the effect of miR-998 on *dCbl* is indirect. For example, miR-998 may function by limiting the level of a positive regulator of *dCbl* expression. However, miR-998 can directly repress dCbl in sensor assays and this repression occurs in a sequence-dependent manner mediated by three different miR-998 target sites in *dCbl*. Similarly, a miR-998 seed family homolog, mir-29 repressed mammalian *c-Cbl* sensors in HeLa cells. Thus, while the mechanism of regulation of Cbl by the mir-998/mir-29 seed family *in vivo* may involve indirect or direct regulation, we favor a model where miR-998 modulates EGFR signaling by directly regulating *dCbl*. Further testing of this model would require introducing mutations in the miR-998 binding sites in the endogenous *dCbl* gene in order to generate *dCbl* alleles that are insensitive to direct targeting by miR-998.

Cbl is a negative regulator of EGFR signaling that binds the activated receptor and induces its ubiquitination and subsequent endocytosis, after which either further downregulation occurs through receptor degradation, or the receptor is retained in endosomes, or recycled back to the plasma membrane [24]. In the eye disc, the transient decrease in EGFR signaling prior to ommatidial specification occurs in part through the sequestration of the EGFR ligand, Spitz, which limits communication from neighbouring cells [13], [41], [42]. Moreover, expression of an EGFR isoform that cannot transmit ligand-initiated signals also stimulated EGFR-dependent cell death in *rbf* mutants [13]. Consistent with this mode of regulation, we showed that changes in the levels of *dCbl*, which limits EGFR signaling from the plasma membrane, modulated E2F-dependent cell death in *rbf* mutants. We suggest that through repression of dCbl expression, miR-998 supported ligand-dependent EGFR signaling in this context, which limited the proapoptotic activity of dE2F1.

miR-998 belongs to the miR-29 seed family of miRNAs, which also includes miR-285 and miR-998 in *Drosophila* [30]. While no mutant phenotypes have been reported, a recent investigation of gain-of-function phenotypes showed similar wing defects caused by overexpression of miR-285 and miR-995, although their molecular basis unknown [43]. Similarly, the functions of miR-29 seed family members cel-miR-49 and cel-miR-83 have not been reported. Seed family members in humans include the less abundant mature miRNA generated from the mir-21 oncomir, mir-21* (mir-21-3p), and mir-593* (mir-593-5p), and miR-29a, miR-29b and miR-29c. Unlike for other seed family members, a number of functions and targets of miR-29 have been reported. While it is not clear whether these functions and targets are common to other seed family members, this possibility warrants further investigation.

In humans, two intergenic mir-29 clusters give rise to three different mature miR-29 miRNAs that share an identical seed sequence, but differ slightly in their 3′ sequences. miR-29 was shown to disrupt epithelial polarity, and cooperate with oncogenic Ras in inducing epithelial-to-mesenchymal transition (EMT) and metastasis through the repression of the tristetraprolin nuclease [44]. Here, we identify a previously unknown miR-29 target, c-Cbl, which functions upstream of Ras in many signaling pathways. This novel link raises the question of whether miR-29 modulates receptor tyrosine kinase-induced EMT. Additional evidence for an oncogenic function of miR-29 came from the identification of PTEN as a miR-29 target [45]. Moreover, the oncogenic viral miRNA BLV-miR-B4 shares the same seed sequence as miR-29 and directly regulates miR-29 targets peroxidasin and HBP1 [46]. However, in different contexts, miR-29 has been shown to function as a tumor suppressor [47]–[49], and miR-29c repressed cancer cell proliferation, and limited E2F activity through the indirect activation of *Rb* [50]. It is not yet clear whether either of the two *mir-29* clusters are transcriptionally regulated by E2F, although the expression of miR-29a is induced in the G1 phase of the cell cycle [51], which is coincident with increased E2F activity.

c-Myc bound both mir-29 cluster gene promoters and repressed miR-29 expression, suggesting that miR-29 is directly regulated by c-Myc [52]. Furthermore, expression of miR-29 lead to a decrease in the expression of E2F targets Cyclin A2, MCM2, and PCNA [52]. It is currently unclear whether the expression of E2F and miR-29 are linked directly, or through c-Myc, which is well-known to induce the expression of E2F1. We note that while miR-998 and miR-29 share at least one common target, their mechanisms of interaction with E2F function may differ. Interestingly, overexpression of dMyc in *Drosophila* embryos lead to both cell death, and decreased expression of miR-998 [53]. It is not known whether miR-998 could block dMyc-induced cell death in *Drosophila* embryos, or whether this would be accomplished through repression of dCbl, and a consequent increase of EGFR signaling.

EGFR/MAPK and Rb/E2F pathways intersect in the regulation of proliferation and cell death and are frequently disrupted in cancer. Our results reveal a novel connection between Rb/E2F and EGFR signaling: miR-998 interacts with both pathways and integrates their activities to effect an overall cellular response. This link may represent an attractive target to intentionally perturb cellular homeostasis thereby sensitizing cells to therapeutic reagents in the treatment of malignancy.

## Materials and Methods

### Fly stocks

All fly crosses were done at 25°C. The following stocks were obtained from Bloomington Drosophila Stock Center at Indiana University: *GMR-Gal4*, *w*; *Dr*/Δ2-3 99B, *Sb*, *dE2f1^EY05005^* (FBal0160590), *dE2f1^7172^* and; *egfr^E1^*. The following stocks were previously published: *UAS-miR-11* (Brennecke et al. 2005), *Act88F-Gal4* and *Act88F-Gal4, UAS-dE2f1* (from Erick Morris and Teiichi Tanimura), *Cbl^F165^*, *FRT 80B*, *UAS-Cbl-L* (A18) and *UAS-Cbl-S* (A1) (from Trudi Schupbach), *rbf1^120a^*, *ey-FLP; act5c>CD2>GAL4, UAS-GFP/CyO, GFP^Act^*, and *rbf1^120a^*, *ey-FLP*/*FM7, GFP^Act^*;; *FRT* 82B, *GFP^Ubi^*, and *rbf1^120a^*, *ey-FLP*/*FM7, GFP^Act^*;; *GFP^Ubi^*, *FRT* 80B (from Nam Sung Moon). *dE2F1* ^Δ*1*^ is a deletion which lacks the genomic region between *P* element insertion *P[XP]E2f^d01508^* and *piggyBac* insertion *PBac[RB]InR^e01952^* and generated according to [54]. *E2f1^d01508^* (FBal0183912), and *InR^d03668^* (FBti0055281) were obtained from the Exelixis collection at Harvard Medical School. Generation of *mir-998^exc222^* mutant allele is described in Protocol S1.

### Construction of UAS-mir-998 transgene

For *in vivo* expression of miR-998, an insert encoding miR-998 was cloned into the pUAST plasmid using standard molecular cloning techniques. This insert contained the *de2f1* intron 5′ sequence flanking two mir-1 chimeras: mir1/mCherry shmiR [55] replaced the *mir-11* gene, and mir-1/998 replaced the *mir-998* gene, which was designed as in Haley et al. (2008) and was synthesized by GenScript USA. Flies carrying UAS-miR-998 were generated by P-element transformation at The Best Gene, Inc.

### Wound healing assay

HeLa cells were seeded in 6-well plates, transfected as described, and cultured until 100% confluent. Straight scratches were made across the cell layer using a 0.2 ml pipette tip. The cells were then gently washed three times with PBS to remove cellular debris and the media was replaced at 0 and 17.5 hours. Photographs of the wound region were taken using a Zeiss AxioObserver A1 microscope and AxioCam IC camera. The wound area was calculated using Image J software.

### 3′ UTR sensor plasmid construction

Sequences were cloned downstream of the Renilla luciferase coding sequence in the psiCheck2 (Promega) plasmid using standard cloning techniques. See Table S6 for sensor sequences.

### Cell culture, transfection and Luciferase assay

HeLa cells were cultured in DMEM+10% FBS, and were transfected with the X-treme Gene HP transfection reagent (Roche) according to the manufacturer's protocol. Cells were harvested 24–48 hours post-transfection. pcDNA3/*mir-998* generated by PCR amplification of the mir-998 gene and standard molecular cloning techniques. pcDNA3/*hsa-mir-29a* was generated by insertion of a GeneString (Invitrogen) with the mir-29a gene sequence in pcDNA3. Insert sequences were verified by sequencing analysis. Sequences are in Table S4. Firefly and Renilla luciferase activity were measured using the Dual Luciferase Assay protocol (Promega).

### Western blot

Antibodies: c-Cbl 1∶200 (sc-170), from Santa Cruz; di-phosphorylated p42/p44 ERK 1∶5000 (M8159) from Sigma-Aldrich; mouse anti-tubulin 1∶10000 (cat# T9026) from Sigma-Aldrich; goat anti-rabbit-HRP (#31460) and goat anti-mouse-HRP (#31430) from Thermo Fisher.

### Immunohistochemistry

Antibodies used were as follows: rabbit anti-C3 (Cleaved Caspase3), lot 26, 1∶75 (Cell Signaling), mouse anti-BrdU 1∶50 (Beckton Dickinson), rat anti-ELAV 1∶50, (Developmental Studies Hybridoma Bank), phosphorylated p42/p44 ERK 1∶200 (M8159) from Sigma-Aldrich, and Cy3-, and Cy5- conjugated anti-mouse, and anti-rabbit secondary antibodies (Jackson Immunoresearch Laboratories). Larval tissues were fixed in 4% formaldehyde in phosphate-buffered saline (PBS) for 30 minutes, permeabilized in 0.3% Triton X-100 in PBS twice for 10 minutes each, blocked in PBS with 0.1% Triton X-100 for 30 minutes at 4°C, and then incubated with antibodies overnight at 4°C in10% normal goat serum, and 0.3% Triton X-100 in PBS. After washing three times for 10 minutes each at room temperature in 0.1% Triton X-100 (in PBS), samples were incubated with appropriate conjugated secondary antibodies for 45 minutes at room temperature in 10% normal goat serum, and 0.3% Triton X-100 (in PBS). After washing with 0.1% Triton X-100 (in PBS), tissues were stored in glycerol+antifade reagents and then mounted on glass slides. To detect S phases, dissected larval eye discs were labeled with BrdU for 2 hrs at room temperature and then fixed overnight in 1.5% formaldehyde, 0.2% Tween 20 in PBS at 4°C. Samples were then digested with DNase (Promega) for 30 minutes at 37°C. Samples were then probed with primary and secondary antibodies as described above. All immunofluorescence was done on a Zeiss Confocal microscope and images were prepared using Adobe Photoshop CS4. All images are confocal single plane images unless otherwise stated as projection images. A minimum of 10 larvae were used for each analysis.

### miRNA target prediction

Comprehensive lists of predicted miR-998 targets and miR-11 targets were compiled from TargetScan [56], MinoTar [57], PITA [58], miRanda [59], and RNAhybrid [60]. Target predictions use for hsa-miR-29 were from TargetScan [61].

### qRT-PCR

Total RNA was isolated from 10 adult heads, 10 larvae, or 30–50 eye discs, with TRIzol (Invitrogen). Reverse transcription to measure standard mRNAs was performed using the iScript kit (BioRad) according to manufacturer's specifications. Quantitative PCR was performed with the SYBR Green I Master (Roche) on a Light Cycler 480 (Roche). miR-11 and miR-998 were measured by Taqman assay (Applied Biosystems). Primer sequences are in Table S5.

### Isolation of *mir-998^exc222^* by *P*-element excision mutagenesis

See Protocol S1.

### Microarray data analysis and enrichment analysis

Microarray gene expression data were analyzed using “Affy” package [62] and differential expression analysis by “Limma” package [63]. Functional and pathway enrichment analysis of differentially expressed genes were counducted using Gitools [64]. See Protocol S1 for detail.

## Supporting Information

Figure S1 miR-11, miR-998 and dE2F1 are co-expressed. (A) cDNA was prepared from RNA extracted from third instar larvae of the genotypes indicated. The expression of *mir-11* and *mir-998*, were measured using qPCR, and normalized to *β-tubulin* and *rp49* levels. A diagram of the *dE2f1* exon/intron structure, mutant alleles, and *mir-11* gene examined is shown. The *dE2f1* ORF corresponds to hatched bars, while untranslated regions are white bars. Introns are represented by horizontal lines. The *dE2f1^07172^ P*-element insertion is 33 nucleotides upstream of the initiator methionine, and the *dE2f1^91^* allele is a C91T point mutation, giving a Q31X early translation termination codon (Mlodzik and Hiromi 1992; Duronio et al. 1995; Brook et al. 1996). (B) Flies carrying the *GMR-Gal4* transgene were crossed to wild-type or *UAS-yki^S168A^* flies. RNA was extracted from third instar larval eye discs, and miR-998, miR-11, *dE2f1*, *β-tubulin*, and *rp49* expression was measured by quantitative RT-PCR. Expression levels shown are relative to *GMR-Gal4*/+.
(TIF)

Figure S2 Overexpression of miR-11, but not miR-998 suppresses *dE2f1*-induced apoptosis in transgenic animals. (A) Flies carrying *Act88F-Gal4* and *UAS-dE2f1 *transgenes were crossed to either a wild-type chromosome (Canton S), *UAS-miR-11*, or *UAS-miR-998*. (B) 3^rd^ instar larval eye discs of indicated genotypes were incubated with BrdU for 90 minutes at room temperature, followed by fixing, and staining with antibodies recognizing BrdU (left), or active caspase (C3) (right). Analysis was performed on a minimum of 10 larvae of each genotype. The position of the morphogenetic furrow is marked with an arrowhead.
(TIFF)

Figure S3 Isolation of a *mir-998* mutant by P-element excision mutagenesis. (A) Three different alleles harboring P-elements near the *dE2f1* gene were initially selected for use in a P-element transposition mutagenesis screen: *InR^d03668^*, *dE2f1^EY05005^*, and *dE2f1^d01508^*. The rate of lethality of transposition events in complementation tests with the *dE2f1^729^* mutant chromosome was compared. *dE2f1^d01508^* had the highest rate of lethality, and was therefore selected for the continuation of the screen. (B) Crossing scheme for the detection of P-element transpositions and complementation test. *dE2f1^d01508^*/Δ2-3, Sb jump start males were crossed to MKRS/TM3, Sb in vials. Individual F1 male progeny with darker eye colour selected from each vial and were crossed to *dE2f1^729^*/TM6B virgin females, and screened for lethality (lack of complementation). (C) Identification of P-element insertion in intron 5. Genomic DNA from *dE2f1^d01508-3928^* was analyzed by PCR using the primer combinations indicated. PCR products in lanes 2 and 3 identified a P-element insertion in intron 5. exon5F1/XP, and XP/exon6R1 would indicate insertion of the P-element in intron 5; and a third PCR reaction using exon5F1/exon6R served as a control for gDNA quality. (D) PCR screening for the presence of *P[XP]^d01508^*. Genomic DNA from *dE2f1^d01508-3928^* was analyzed by PCR using the primer combinations indicated.
(TIF)

Protocol S1 Generation of *mir-998* mutant and bioinformatics analysis of Affymetrix microarrays.
(DOCX)

Table S1 The complied list of predicted miR-11, miR-998 and miR-29 targets.
(XLSX)

Table S2 GOBP enrichment analysis of DE genes in *mir-998* mutants.
(XLSX)

Table S3 Compiled list of genes related to EGFR pathway.
(XLSX)

Table S4 Sequence of miRNA-expressing constructs.
(XLSX)

Table S5 Primers sequence used in this study.
(XLSX)

Table S6 Sequence inserted in 3′UTR luciferase sensor reporters.
(XLSX)
