# Supplementary figures and images for: An Intronic microRNA Links Rb/E2F and EGFR Signaling
Source: PLoS Genet. 2014 Jul 24;10(7):e1004493. doi: 10.1371/journal.pgen.1004493 (PMC4109884; doi:10.1371/journal.pgen.1004493)

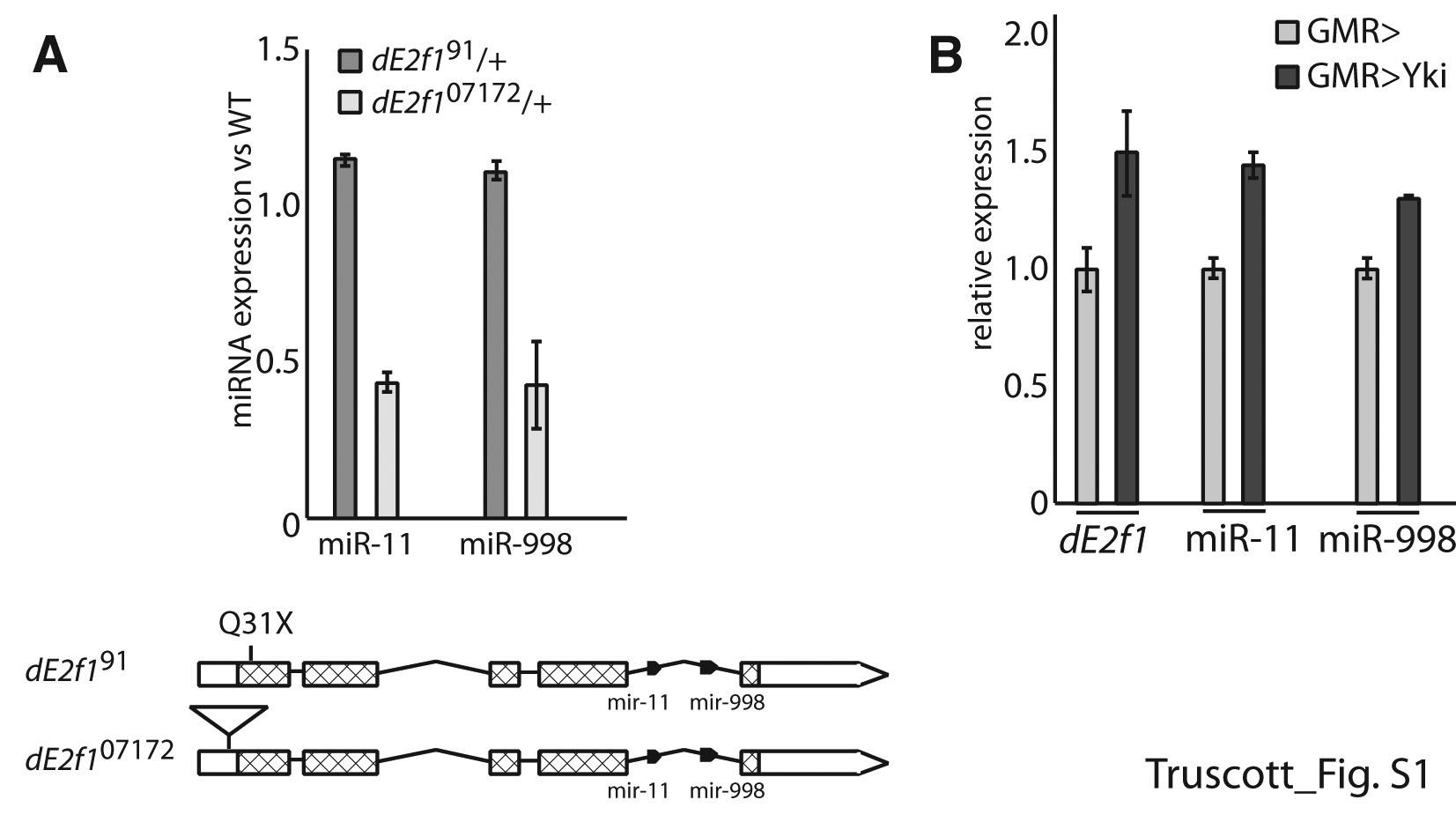

Supplement: Figure S1 — miR-11, miR-998 and dE2F1 are co-expressed. (A) cDNA was prepared from RNA extracted from third instar larvae of the genotypes indicated. The expression of mir-11 and mir-998, were measured using qPCR, and normalized to β-tubulin and rp49 levels. A diagram of the dE2f1 exon/intron structure, mutant alleles, and mir-11 gene examined is shown. The dE2f1 ORF corresponds to hatched bars, while untranslated regions are white bars. Introns are represented by horizontal lines. The dE2f107172 P-element insertion is 33 nucleotides upstream of the initiator methionine, and the dE2f191 allele is a C91T point mutation, giving a Q31X early translation termination codon (Mlodzik and Hiromi 1992; Duronio et al. 1995; Brook et al. 1996). (B) Flies carrying the GMR-Gal4 transgene were crossed to wild-type or UAS-ykiS168A flies. RNA was extracted from third instar larval eye discs, and miR-998, miR-11, dE2f1, β-tubulin, and rp49 expression was measured by quantitative RT-PCR. Expression levels shown are relative to GMR-Gal4/+. (TIF) [file pgen.1004493.s001.tif]

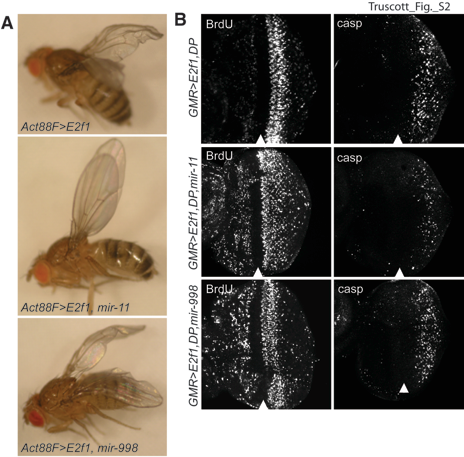

Supplement: Figure S2 — Overexpression of miR-11, but not miR-998 suppresses dE2f1-induced apoptosis in transgenic animals. (A) Flies carrying Act88F-Gal4 and UAS-dE2f1 transgenes were crossed to either a wild-type chromosome (Canton S), UAS-miR-11, or UAS-miR-998. (B) 3rd instar larval eye discs of indicated genotypes were incubated with BrdU for 90 minutes at room temperature, followed by fixing, and staining with antibodies recognizing BrdU (left), or active caspase (C3) (right). Analysis was performed on a minimum of 10 larvae of each genotype. The position of the morphogenetic furrow is marked with an arrowhead. (TIFF) [file pgen.1004493.s002.tiff]

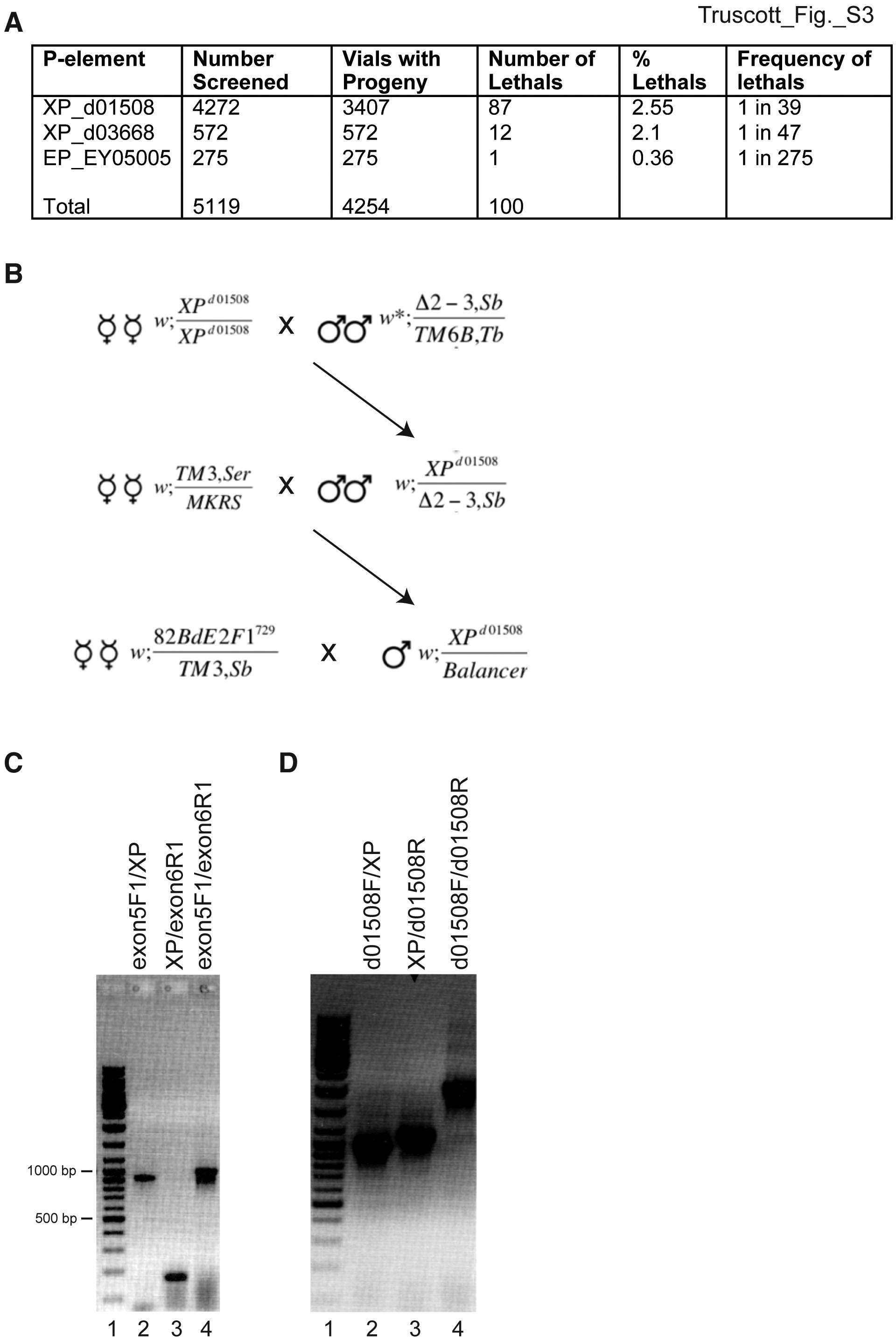

Supplement: Figure S3 — Isolation of a mir-998 mutant by P-element excision mutagenesis. (A) Three different alleles harboring P-elements near the dE2f1 gene were initially selected for use in a P-element transposition mutagenesis screen: InRd03668, dE2f1EY05005, and dE2f1d01508. The rate of lethality of transposition events in complementation tests with the dE2f1729 mutant chromosome was compared. dE2f1d01508 had the highest rate of lethality, and was therefore selected for the continuation of the screen. (B) Crossing scheme for the detection of P-element transpositions and complementation test. dE2f1d01508/Δ2-3, Sb jump start males were crossed to MKRS/TM3, Sb in vials. Individual F1 male progeny with darker eye colour selected from each vial and were crossed to dE2f1729/TM6B virgin females, and screened for lethality (lack of complementation). (C) Identification of P-element insertion in intron 5. Genomic DNA from dE2f1d01508-3928 was analyzed by PCR using the primer combinations indicated. PCR products in lanes 2 and 3 identified a P-element insertion in intron 5. exon5F1/XP, and XP/exon6R1 would indicate insertion of the P-element in intron 5; and a third PCR reaction using exon5F1/exon6R served as a control for gDNA quality. (D) PCR screening for the presence of P[XP]d01508. Genomic DNA from dE2f1d01508-3928 was analyzed by PCR using the primer combinations indicated. (TIF) [file pgen.1004493.s003.tif]
