# Supplementary material for: An Intronic microRNA Links Rb/E2F and EGFR Signaling
Source: PLoS Genet. 2014 Jul 24;10(7):e1004493. doi: 10.1371/journal.pgen.1004493 (PMC4109884; doi:10.1371/journal.pgen.1004493)
Supplement: Protocol S1 — Generation of mir-998 mutant and bioinformatics analysis of Affymetrix microarrays. (DOCX) [file pgen.1004493.s004.docx]

**PROTOCOL S1**

**Generation of *mir-998* mutant by P-element imprecise excision**

*dE2f1^d01508^*/Δ2-3, Sb jump start males were crossed to MKRS/TM3, Sb in vials. Individual F1 male progeny with darker eye colour selected from each vial and were crossed to *dE2f1^729^*/TM6B virgin females for identification of putative transposition events based on lack of complementation. However, the P[XP] transposon contains the mini-white gene, and its stop codon could permit the expression of a truncated dE2F1 protein, which may not be lethal. In order to detect insertions that generated viable *dE2f1* alleles, we performed PCR analysis in parallel. After 5 days, the F1 males were removed and genomic DNA was extracted from pools of 20 flies according to standard methods. Two different PCR reactions were performed to screen for the insertion of P-elements in intron 5 of the *dE2f1* gene: PCR products from exon5F1/XP, and XP/exon6R1 would indicate insertion of the P-element in intron 5. Of 5119 transposition events screened, 100 alleles that did not complement the *dE2f1^729^* null mutation were identified, from which only one transpostion into intron 5 was detected. Insertion occurred within the *mir-998* gene at 3R:17,447,644..17,447,645, which interrupted the stem-loop and prevented the expression of mature miR-998. A second round of P-element transposition was performed to screen for the precise loss of the original P-element, *P[XP]^d01508^*, which was verified by sequencing.

A final round of P-element transposition was performed to screen for imprecise excision of the P-element from *mir-998*, that specifically disrupted the *mir-998* gene, but did not affect *mir-11* and *dE2f1*. Imprecise excision events that were detectable with the exon5F1/exon6R primer set occurred at a frequency of approximately 0.5%. Of >400 excision events, we did not identify any small deletions by PCR with primers against the 5’ and 3’ ends of intron 5. However, we identified two independent imprecise excisions in which a transposition-defective P-element remained within the sequence of the *mir-998* gene. The allele *mir-998^exc222^* was a partial excision that retained 87 bp, including the 8 bp target duplication, a partial, and an intact inverted terminal repeat, as well as 44bp P[XP] sequence.

**Drosophila Microarray Analysis**

**RNA isolation and Hybridization**

Eye imaginal disc total RNA was isolated with TRIzol reagent (Invitrogen) and further purified using the RNeasy kit (Qiagen). In accordance with the Affymetrix protocol (Affymetrix expression manual), all samples were processed and a total of 15ug of fragmented and labeled cRNA were hybridized to the Affymetrix GeneChip arrays (Drosophila genome 385K 2.0). The Chips were then washed and stained using an Affymetrix Fluidics Station 450 and Flourescence was detected using the Affymetrix GS3000.

**Microarray data analysis**

Affymatrix CEL files were background corrected, normalized using Bioconductor package “affy” (Gentleman et al., 2004) (Gautier et al., 2004) (version 1.28.1) using 'rma' algorithm. Quality of microarray experiment (data not shown) was verified by Bioconductor package “arrayQualityMetrics” (Kauffmann et al., 2009) (version 3.2.4 under Bioconductor version 2.7; R version 2.12.1). To determine genes that are differentially expressed (DE) between two experimental condition, Bioconductor package Limma (Smyth, 2004) was utilized to generate contrast matrices and fit the corresponding linear model. Probe annotations to Ensembl gene ID, gene symbol and description, we use the annotation description from affymetrix website (<http://www.affymetrix.com/support/technical/annotationfilesmain.affx>), Biomart, Ensembl v.55 (*Drosophila melanogaster* genes; BDGP 5.4) (Hubbard et al., 2007) and FlyBase database (DrysdaleFlyBase Consortium, 2008). When more than one probes were annotated to same gene, highest absolute expression value was considered (maximizing). To consider a gene is differentially expressed, multiple test corrected, FDR (Benjamini and Hochberg, 1995) p-value ≤ 0.05 was used as as cut off. The data discussed in this publication have been deposited in NCBI's Gene Expression Omnibus (Edgar et al., 2002) and are accessible through GEO Series accession number GSE51724 ([http://www.ncbi.nlm.nih.gov/geo/query/acc.cgi?acc=GSE51724](http://www.ncbi.nlm.nih.gov/geo/query/acc.cgi?acc=GSE51724))

**Functional Enrichment Analysis**

Functional annotation of differentially target genes is based on Gene Ontology (GO) (Ashburner et al., 2000) ([http://www.geneontology.org](http://www.geneontology.org/#_blank)) as extracted from EnsEMBL (Hubbard et al., 2007)

Accordingly, all genes are classified into the ontology categories biological process (GOBP) and pathways when possible. We have taken only the GO/pathway categories that have 20-120 genes annotated. We used Gitools for enrichment analysis and heatmap generation (Perez-Llamas and López-Bigas, 2011); [www.gitools.org](http://www.gitools.org/#_blank)). Resulting p-values were adjusted for multiple testing using the Benjamin and Hochberg's method of False Discovery Rate (FDR) (Benjamini and Hochberg, 1995).

**References:**

Ashburner, M., Ball, C.A., Blake, J.A., Botstein, D., Butler, H., Cherry, J.M., Davis, A.P., Dolinski, K., Dwight, S.S., Eppig, J.T., Harris, M.A., Hill, D.P., Issel-Tarver, L., Kasarskis, A., Lewis, S., Matese, J.C., Richardson, J.E., Ringwald, M., Rubin, G.M., Sherlock, G., 2000. Gene ontology: tool for the unification of biology. The Gene Ontology Consortium. Nat Genet 25, 25–29.

Benjamini, Y., Hochberg, Y., 1995. Controlling the false discovery rate: a practical and powerful approach to multiple testing. Journal of the Royal Statistical Society. Series B (Methodological) 289–300.

Drysdale, R., FlyBase Consortium, 2008. FlyBase : a database for the Drosophila research community. Methods Mol Biol 420, 45–59.

Edgar, R., Domrachev, M., Lash, A.E., 2002. Gene Expression Omnibus: NCBI gene expression and hybridization array data repository. Nucleic Acids Res 30, 207–210.

Gautier, L., Cope, L., Bolstad, B.M., Irizarry, R.A., 2004. affy--analysis of Affymetrix GeneChip data at the probe level. Bioinformatics 20, 307–315.

Gentleman, R.C., Carey, V.J., Bates, D.M., Bolstad, B., Dettling, M., Dudoit, S., Ellis, B., Gautier, L., Ge, Y., Gentry, J., Hornik, K., Hothorn, T., Huber, W., Iacus, S., Irizarry, R., Leisch, F., Li, C., Maechler, M., Rossini, A.J., Sawitzki, G., Smith, C., Smyth, G., Tierney, L., Yang, J.Y.H., Zhang, J., 2004. Bioconductor: open software development for computational biology and bioinformatics. Genome Biol 5, R80.

Hubbard, T.J.P., Aken, B.L., Beal, K., Ballester, B., Caccamo, M., Chen, Y., Clarke, L., Coates, G., Cunningham, F., Cutts, T., Down, T., Dyer, S.C., Fitzgerald, S., Fernandez-Banet, J., Graf, S., Haider, S., Hammond, M., Herrero, J., Holland, R., Howe, K., Howe, K., Johnson, N., Kahari, A., Keefe, D., Kokocinski, F., Kulesha, E., Lawson, D., Longden, I., Melsopp, C., Megy, K., Meidl, P., Ouverdin, B., Parker, A., Prlic, A., Rice, S., Rios, D., Schuster, M., Sealy, I., Severin, J., Slater, G., Smedley, D., Spudich, G., Trevanion, S., Vilella, A., Vogel, J., White, S., Wood, M., Cox, T., Curwen, V., Durbin, R., Fernandez-Suarez, X.M., Flicek, P., Kasprzyk, A., Proctor, G., Searle, S., Smith, J., Ureta-Vidal, A., Birney, E., 2007. Ensembl 2007. Nucleic Acids Res 35, D610–7.

Kauffmann, A., Gentleman, R., Huber, W., 2009. arrayQualityMetrics—a bioconductor package for quality assessment of microarray data. Bioinformatics 25, 415–416.

Perez-Llamas, C., López-Bigas, N., 2011. Gitools: analysis and visualisation of genomic data using interactive heat-maps. PLoS ONE 6, e19541.

Smyth, G.K., 2004. Linear models and empirical bayes methods for assessing differential expression in microarray experiments. Stat Appl Genet Mol Biol 3, Article3.
